# The Mediating Effect of Regulatory Emotional Self-Efficacy on the Association between Self-Esteem and School Bullying in Middle School Students: A Cross-Sectional Study

**DOI:** 10.3390/ijerph15050991

**Published:** 2018-05-15

**Authors:** Xiaoqin Wang, Yue Zhang, Zhaozhao Hui, Wanyue Bai, Paul D. Terry, Mei Ma, Yang Li, Li Cheng, Wei Gu, Mingxu Wang

**Affiliations:** 1 Department of Public Health, Xi’an Jiaotong University Health Science Center, Xi’an 710061, China; wangxiaoqin@mail.xjtu.edu.cn (X.W.); zymoon95@126.com (Y.Z.); huizhaozhao93@163.com (Z.H.); leeyangcherry@163.com (Y.L.); 232guwei@mail.xjtu.edu.cn (W.G.); 2 Xi’an Tieyi High School, Xi’an 710061, China; baiwanyue17@163.com; 3 Department of Medicine, Graduate School of Medicine, University of Tennessee Medical Center, Knoxville, TN 37920, USA; pterry@utmck.edu; 4 Nursing Department, Xian Yang Central Hospital, Xianyang 712000, China; wysun201314195@163.com; 5 Faculty of Medicine, The Chinese University of Hong Kong, Shatin 999077, Hong Kong, China; lilycheng@link.cuhk.edu.hk

**Keywords:** school bullying, self-esteem, regulatory emotional self-efficacy, mediating effect

## Abstract

School bullying is negatively associated with self-esteem, but psychological mediators of bullying have yet to be clarified. We examined regulatory emotional self-efficacy (RESE) as a possible mediator in the association between self-esteem and school bullying. A cross-sectional study of 995 adolescents was conducted in two middle schools of Xi’an. All of the participants completed the Chinese version of the School Bullying Experience Questionnaire (C-SBEQ), Self-Esteem Scale (SES), and Regulatory Emotional Self-Efficacy Scale (RESE). Descriptive statistics analysis, the bias corrected percentile Bootstrap CI method, and structural equation modelling were used to analyze the data. The results showed that 418 students (42.0%) reported that they were involved in school bullying in the past year. Self-esteem was negatively associated with school bullying (total effect: β = −0.275, 95% CI = −0.381–−0.034), and RESE mediated the association between self-esteem and school bullying (indirect effect: β = −0.136, 95% CI = −0.245–−0.037). Furthermore, self-esteem had an indirect effect through perceived self-efficacy in managing negative affect, while self-esteem had no indirect effect through self-efficacy in the expression of positive affect. The present study suggests that school authorities and the related education departments should not only focus on improving students’ self-esteem, but should also pay more attention to students’ RESE, in order to mitigate, and potentially reduce, the occurrence of bullying.

## 1. Introduction

School bullying is a public health problem because of the high prevalence and its consequences for those that are involved [1]. A recent meta-analysis [2] reported that the mean prevalence rate of bullying for perpetration was 35% and for victimization was 36% across 80 studies. In China, a recent review reported that the prevalence of bullying perpetration and victimization ranged from 2% to 34%, and 2% to 66%, respectively [3]. Most importantly, the consequences of bullying for the victims, but also for the perpetrators, can be severe and long-lasting. For the perpetrators, school bullying might result in even criminality or antisocial behavior [4], and it was found to be a predictor of delinquency [5], problems adapting to the demands of adulthood (e.g., housing problems, relationship problems, employment problems, involvement in fights, drug and alcohol abuse), and other social and economic problems [6] in adulthood. For the victims, school bullying may cause multiple physical and psychological health consequences [7], such as lower academic achievement [8], pain-related complaints [9], anxiety, depression [10], subclinical psychotic experiences [11], self-injurious behaviors [12], and even suicidal ideation and suicide attempts [13].

Self-esteem is likely to play an important role in children’s development, and is a large part of adolescents’ self-understanding [14,15]. Low levels of self-esteem have been related to aggressive behavior, antisocial behavior, and depression [16,17]. There has been a considerable body of research reporting the association between self-esteem and school bullying [18,19,20,21]. A recent meta-analysis [14] found that self-esteem was negatively associated with both peer victimization and bullying perpetration. However, studies that address explanatory components of this association have been scarce. One study reported that the association between self-esteem and bullying perpetration was moderated by the perceived school climate (unfriendly, unfair, and nonsupportive) [20].

According to Bandura’s self-efficacy theory, self-efficacy refers to the belief in one’s ability to complete the activity related to that competency. Self-efficacy is one of the most important preconditions of behavior change [22,23], and positive self-esteem presupposes the corresponding self-efficacy [24]. Previous studies have reported that reducing violent behavior and aggressive behavior among adolescents requires a strong sense of self-efficacy [25], which will also decrease the likelihood of being a victim [26]. In addition, school bullying was associated with emotion regulation [27]. Regulatory emotional self-efficacy (RESE) is a specific aspect of self-efficacy that entails a subjective self-appraisal of one’s emotional competence in emotion regulation [28], and it reflects one’s confidence of his/her own competence in emotion regulation [29]. Therefore, high levels of RESE may be associated with bullying behavior. In addition, previous studies have demonstrated that RESE contributed to preventing delinquent behavior and encouraging one’s pro-social behavior [30,31], and it was shown to reduce violent and aggressive behaviors [25]. Regarding the association between self-esteem and RESE, self-esteem may be a major lens through which he/she views and evaluates his/her experience in the world, and thus his/her feeling of competence [32]. An association between self-esteem and RESE was examined in a longitudinal study, in which subjects with high self-esteem perceived a higher degree of self-efficacy in expressing positive affect and regulating negative affect than people with low self-esteem [33]. Taken together, we would take RESE as a possible mediator between self-esteem and school bullying.

The present study aims to examine the associations among self-esteem, RESE, and school bullying, in particular, whether RESE mediates the association between self-esteem and school bullying. We hypothesized that self-esteem could influence school bullying, and this association might be mediated by RESE.

## 2. Materials and Methods

### 2.1. Participants and Procedure

A cross-sectional survey was conducted in Xi’an, China. A convenience sampling method was used to select participants (aged 11–18 years) in two middle schools of Xi’an. We enrolled the students of 20 classes in the Second Affiliated Middle School of Xi’an Jiaotong University and four classes in Xi’an Tieyi High School. All of the participants signed consent forms. A total of 1050 questionnaires were issued and 995 were recovered, with an effective recovery rate of 94.8%. The study was approved by the Biomedical Ethics Committee of Xi’an Jiaotong University Health Science Center (Ethical code: 2016-256).

### 2.2. Instruments

#### 2.2.1. Demographic Variables and Background Characteristics

Demographic variables included school, age, gender, grade, father’s educational background, mother’s educational background, and parental marital status. Background characteristics included the availability of pocket money, academic achievement, personality type (introverted, outgoing, somewhere in between, or not sure), living condition (living with parents, grandparents, or others), tobacco smoking, and alcohol consumption.

#### 2.2.2. The Chinese Version of the School Bullying Experience Questionnaire (C-SBEQ)

The C-SBEQ scale was used to evaluate the prevalence of school bullying in the past year [34]. This scale consists of 16 items and four subscales. Items 1 to 4 evaluate the consequences of being a victim of passive bullying, including social exclusion, being called names, and otherwise being spoken ill to. Items 5 to 8 evaluate the consequences of being a victim of active bullying, including being beaten, being extorted for money, being made to do work, and having school supplies and snacks taken away. Items 9 to 12 evaluate the consequences of being a perpetrator of passive bullying. Items 13 to 16 evaluate the consequences of being a perpetrator of active bullying. The frequency of school bullying was assessed with the categories never (0), just a little (1), often (2), and all the time (3). Participants who chose answer 2 or answer 3 for any item were identified as victims or perpetrators. A previous study validated the psychological components of the questionnaire in Chinese middle school students with the test-retest reliability of these subscales ranging from 0.742 to 0.813 [34]. The Cronbach’s α coefficient in our study was 0.800.

#### 2.2.3. The Self-Esteem Scale (SES)

The SES was used to assess each participant’s level of self-esteem [35]. The SES contains 10 items, five of which assess level of positive self-esteem, and five evaluate level of negative self-esteem. Each item is scored from 1 (completely disagree) to 4 (completely agree). A previous study reported that the internal consistency reliability of the SES for Chinese participants was 0.84 [36]. The Cronbach’s α coefficient in our study was 0.775.

#### 2.2.4. Regulatory Emotional Self-Efficacy Scale (RESE)

The RESE was used to assess each participant’s perceived capability to successfully manage his or her emotional life [37]. RESE has 12 items, with scores ranging from 1 (completely disagree) to 5 (completely agree). This scale has two subscales, perceived self-efficacy in expressing positive affect (POS) (four items) and perceived self-efficacy in managing negative affect (NEG) (eight items). Further, the NEG subscale was made up of two lower-order subscales of perceived self-efficacy in managing despondency-distress (DES) (four items), and anger-irritation (ANG) (four items). A previous study [38] in China found acceptable test-retest, internal reliability, and validity values of all subscales among middle school students (Cronbach’s α = 0.79, test-retest coefficient = 0.87). The Cronbach’s α coefficient in our study was 0.886.

### 2.3. Statistical Analysis

**Figure 1:**
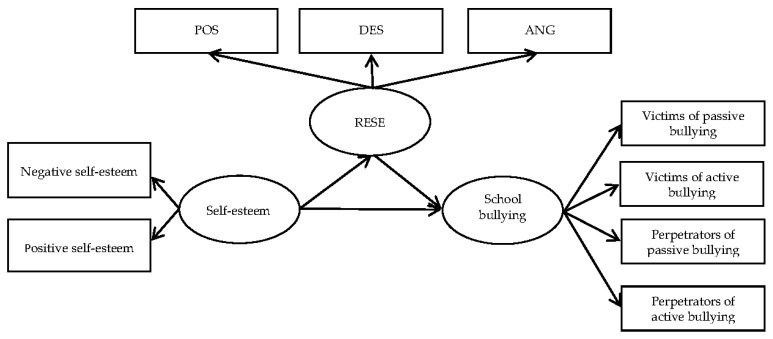
Structural model of regulatory emotional self-efficacy (RESE) as a mediator of self-esteem and school bullying. All three variables (school bullying experience, self-esteem and RESE) are latent variables measured by a few indicators. POS = the perceived capability of expressing positive affect, DES = the perceived capability of managing despondency-distress, ANG = the perceived capability of managing anger-irritation.

Data management and data analysis were performed using Epidata (The Epidata Association, Odense, Denmark) Version 3.1, SPSS (Statistical Package for the Social Sciences for Windows, IBM, Armonk, NY, USA) Version 22.0 and AMOS (Analysis of Moment Structures, IBM, Armonk, NY, USA) version 21.0 Descriptive statistics were used to describe participants’ demographic and background factors, as well as the prevalence of school bullying. Structural equation modelling was used to examine the mediating effect of RESE on the association between self-esteem and school bullying. The bias corrected percentile Bootstrap CI method was used to calculate the 95% confidence intervals (95% CIs) for the coefficients for the total, direct, and indirect effects. Coefficients were considered to be statistically significant if the 95% CIs did not cross zero [39]. Statistical significance was set at *p <* 0.05; all the tests were two-sided. The theoretical model is shown in Figure 1.

## 3. Results

### 3.1. The Demographic Characteristics and Prevalence of School Bullying

The final sample included 995 middle school students. There were 541 (54.4%) males and 454 (45.6%) females that were aged 11 to 18 years (14.2 ± 1.6). Of the participants, 418 (42.0%) reported having been involved in school bullying. In addition, 21.1% of the participating students were only victims, 3.3% of them were only perpetrators, and 17.6% of them were victim-perpetrators. The average self-esteem score of the participants was 29.2 ± 4.7 and the average RESE score was 44.1 ± 8.8.

### 3.2. The Mediating Effect of RESE

**Figure 2:**
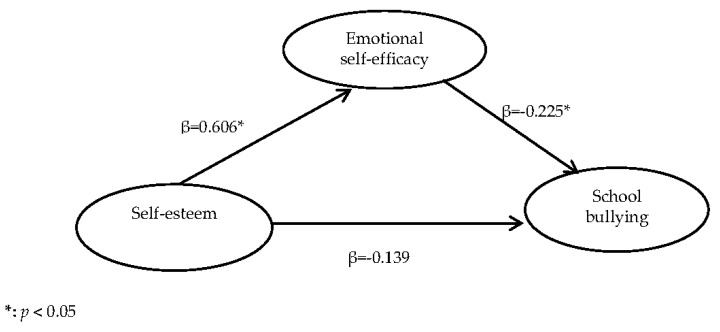
The mediation model of RESE (the SEM provided acceptable indices of goodness-of-fit, GFI = 0.966, CFI = 0.942, RMSEA = 0.075. The path weights in the graph were standardized.).

The mediation model with standardized regression coefficients is shown in Figure 2. Indices of goodness-of-fit of the model showed a desirable fit, with GFI = 0.966, CFI = 0.942, and RMSEA = 0.075. The GFI and CFI exceeded the recommended value of 0.90 [40,41], and the RMSEA less than 0.08 [41,42] was acceptable. The squared multiple correlation was 10.8%. Self-esteem was significantly associated with RESE (β = 0.606, 95% CI = 0.467–0.730, *p* < 0.001). RESE was significantly associated with school bullying experience (β = −0.225, 95% CI = −0.381–−0.034, *p* = 0.027). After controlling for RESE, there was no significant direct effect between self-esteem and school bullying (β = −0.139, 95% CI = −0.405–0.020, *p* = 0.091), indicating the possibility of one or more mediating effects. The result of Bootstrap analyses further indicated a mediating effect of RESE. Self-esteem had a significant total effect on school bullying (total effect: β = −0.275, 95% CI = −0.436–−0.135), and the association between self-esteem and school bullying was mediated by RESE (indirect effect: β = −0.136, 95% CI = −0.245–−0.037).

In analyses exploring the mediating effect of RESE on the association between self-esteem and only bullying victimization, only bullying perpetration, and the overlap of perpetration and victimization, the results showed that self-esteem had indirect effects on only bullying perpetration (indirect effect: β = −0.070, 95% CI = −0.105–−0.041) and overlap of perpetration and victimization (indirect effect = −0.022, 95% CI = −0.040–−0.006) through RESE, but no indirect effect on only bullying victimization through RESE.

We used multiple mediation modes to test the mediating effect of every dimension of RESE, which means the mediating effect of one mediating variable after controlling for other variables (Table 1). The results indicated that DES mediated the association between self-esteem and bullying in both the victims of passive bullying and the perpetrators of passive bullying. Similarly, ANG had the mediating effect on the association between self-esteem and bullying in both the perpetrators of passive bullying and the perpetrators of active bullying. POS had no mediating effect.

## 4. Discussion

Our study found a high prevalence of school bullying among middle school students. Moreover, we found that RESE had a mediating effect in the association between self-esteem and school bullying. Especially, self-esteem had indirect effects on only bullying perpetration and the overlap of perpetration and victimization through RESE, but no indirect effect on only bullying victimization through RESE. We also found that NEG had a mediating effect, whereas POS had no mediating effect.

We found that 42.0% of the participating students in our study had been involved in school bullying, with 38.7% being reported being victims and 20.9% perpetrators. We found that 21.1% of the students were only victims, 3.3% of the students were only perpetrators, and 17.6% of the students were victim-perpetrators. In a recent study that examined the prevalence of bullying among adolescents from all types of pre-colleges in China, the results showed that the prevalence of only victims, only perpetrators, and victim-perpetrators was 18.7%, 1.7%, and 7.4%, respectively [43]. When compared with this study, our results showed higher prevalence of any type of bullying, which indicated that middle school students may be particularly vulnerable to problems that are associated with school bullying.

Although there has been no previous research examining the mediating effect of RESE on the association between self-esteem and school bullying, two studies on RESE have shown the mediating effect of RESE on other forms of risk behaviors [38,44]. One study examined the mediating effect of RESE on the associations among paternal, maternal, and peer attachment and internalizing symptoms, in which it was reported that RESE mediated the association between all types of attachment and internalizing symptoms [44]. Internalizing symptoms and externalizing symptoms (e.g., aggression) commonly co-occur [45,46], and in most cases, the perpetration of active bullying is externalized aggression. Another study examined the association among social anxiety, aggression, depression, and RESE, and reported that RESE mediated the association between social anxiety and aggression [39], which is a part of school bullying.

Our findings are in accordance with our view that self-esteem can influence school bullying for only perpetrators and victim-perpetrators through RESE. Positive self-esteem presupposes the corresponding self-efficacy [24], and according to Bandura’s theory, self-efficacy determines whether the subjects can make good use of their relative competencies [22]. Our finding concerning the mediating effect of RESE corroborated and extended the results of previous studies that found a significant effect of self-esteem on school bullying [14,18]. Our findings also suggest that students with a low-level of self-esteem engage in more bullying behaviors may be due to their low level of RESE. Therefore, RESE may be an important component in the association of self-esteem with school bullying.

We found that RESE had a mediating effect on the association between self-esteem and only bullying perpetration and overlap victimization and perpetration, but had no mediating effect on the association between self-esteem and only bullying victimization. Studies concerning the mediating effect on the association between self-esteem and only bullying victimization, only bullying perpetration and overlap victimization and perpetration have not been retrieved. A previous study has demonstrated that RESE can reduce aggressive behaviors [25], which include one type of perpetration [47]. The reason why RESE mediated overlap victimization and perpetration but did not mediate only victimization needs further research. This finding suggests that we might make strategies to improve their level of RESE, aiming at the groups of perpetrators and the victim-perpetrators with low self-esteem.

We also found that self-esteem had an indirect effect on school bullying through NEG while self-esteem had no indirect effect on school bullying through POS. This finding may have several explanations. First, the NEG predicted the emotional stability and POS was not related to emotional stability [48]. Then, ANG was especially associated with aggression, while POS was associated with pro-social behavior [37]. Moreover, externalizing symptoms (like aggression) were associated with the cognition and capability of managing negative affect [31], rather than positive affect, thereby making it likely that NEG had mediating effect while POS had no mediating effect. Thus, NEG may be an important consideration when implementing interventions to reduce school bullying.

There are three limitations in the present study. First, this study was cross-sectional in design and cannot establish a causal relationship. For example, the lack of follow-up precludes determining whether bullying preceded or succeeded the aspects of self-esteem that we measured. Second, only self-reported values were used to evaluate self-esteem, RESE and school bullying, raising the possibility of measurement error. Finally, this study was only conducted among students who were attending school during the investigation period. Student absence from school might be associated with school bullying, which (if so) might have biased our results.

## 5. Conclusions

Our study shows an association between self-esteem and school bullying, with a mediating role of RESE, which provides new evidence about how self-esteem influences school bullying. Our results suggest that school authorities and related education departments can take steps to enhance students’ RESE, especially for perpetrators and victim-perpetrators. If confirmed in future studies, the mediating role of RESE in school bullying would suggest both prevention and coping strategies for middle school students.

## Figures and Tables

**Table 1:** The mediating effect of RESE on the association between self-esteem and school bullying

Indirect Effect	β	Boot SE	95% CI
From positive self-esteem to victims of passive bullying via RESE	−0.042	0.018	−0.081–−0.009
via POS	0.004	0.015	−0.026–0.031
via DES	−0.038 *	0.016	−0.069–−0.009
via ANG	−0.008	0.010	−0.029–0.011
From positive self-esteem to victims of active bullying via RESE	−0.018	0.012	−0.048–0.003
via POS	−0.010	0.009	−0.028–0.006
via DES	−0.003	0.008	−0.021–0.012
via ANG	−0.005	0.006	−0.018–0.006
From positive self-esteem to perpetrators of passive bullying via RESE	−0.046	0.016	−0.080–−0.016
via POS	0.003	0.013	−0.024–0.028
via DES	−0.029 *	0.012	−0.053–−0.005
via ANG	−0.020 *	0.010	−0.040–−0.003
From positive self-esteem to perpetrators of active bullying via RESE	−0.019	0.015	−0.050–0.006
via POS	−0.004	0.011	−0.027–0.016
via DES	0.001	0.009	−0.017–0.019
via ANG	−0.015 *	0.007	−0.032–−0.003
From negative self-esteem to victims of passive bullying via RESE	0.020	0.007	0.007–0.037
via POS	0.002	0.004	−0.005–0.012
via DES	0.015 *	0.007	0.004–0.032
via ANG	0.003	0.004	−0.002–0.012
From negative self-esteem to victims of active bullying via RESE	0.006	0.004	−0.001–0.018
via POS	0.004	0.003	−0.001–0.011
via DES	0.001	0.003	−0.005–0.008
via ANG	0.002	0.002	−0.001–0.008
From negative self-esteem to perpetrators of passive bullying via RESE	0.017	0.007	0.006–0.033
via POS	<0.001	0.004	−0.007–0.008
via DES	0.011 *	0.005	0.003–0.024
via ANG	0.006 *	0.004	0.001–0.017
From negative self-esteem to perpetrators of active bullying via RESE	0.007	0.005	−0.001–0.019
via POS	0.002	0.003	−0.003–0.010
via DES	<0.001	0.003	−0.007–0.007
via ANG	0.005 *	0.003	0.001–0.013

* *p* < 0.05; controlled for school, age, gender, grade, father’s educational background, mother’s educational background, parental marital status, the availability of pocket money, academic achievement, personality type, living condition, tobacco smoking, and alcohol consumption.
